# Recent Advances in Cellulose Nanofiber Modification and Characterization and Cellulose Nanofiber-Based Films for Eco-Friendly Active Food Packaging

**DOI:** 10.3390/foods13243999

**Published:** 2024-12-11

**Authors:** Jiaojiao Sun, Xi Yang, Yifan Bai, Zhisheng Fang, Shuai Zhang, Xiaoyu Wang, Yali Yang, Yurong Guo

**Affiliations:** 1 Engineering Research Center for High-Valued Utilization of Fruit Resources in Western China, Ministry of Education, Shaanxi Normal University, 620 West Changan Avenue, Xi’an 710119, China; 18706874592@163.com (J.S.); wangxiaoyu@snnu.edu.cn (X.W.); yangyali@snnu.edu.cn (Y.Y.); 2 National Research & Development Center of Apple Processing Technology, Shaanxi Normal University, 620 West Changan Avenue, Xi’an 710119, China; 3 College of Food Engineering and Nutritional Science, Shaanxi Normal University, 620 West Changan Avenue, Xi’an 710119, China; 4 School of Electronic Engineering, Xi’an University of Posts and Telecommunications, Xi’an 710121, China; byf@stu.xupt.edu.cn (Y.B.); 3076309319@stu.xupt.edu.cn (Z.F.); 5 College of Food Science and Engineering, Ningbo University, Ningbo 315100, China; yangxib16224@163.com

**Keywords:** cellulose nanofibers (CNFs), modification, characterization, eco-friendly food packaging

## Abstract

There is growing interest in the use of bio-based materials as viable alternatives to petrochemical-based packaging. However, the practical application of bio-based films is often hampered by their poor barrier and poor mechanical properties. In this context, cellulose nanofibers (CNFs) have attracted considerable attention owing to their exceptional biodegradability, high aspect ratio, and large surface area. The extraction of CNFs from agricultural waste or non-food biomass represents a sustainable approach that can effectively balance cost and environmental impacts. The functionalization of CNFs improves the economics of raw materials and production processes while expanding their applications. This paper reviews recent advances in cellulose nanofibers, including their sources, surface modification, and characterization techniques. Furthermore, we systematically discuss the interactions of CNFs with different composites in the development of functional food films. Finally, we highlight the application of cellulose nanofiber films in food preservation. Due to their environmentally friendly properties, CNFs are a promising alternative to petroleum-based plastics. The aim of this paper is to present the latest discoveries and advances in CNFs while exploring the future prospects for edible food films, thereby encouraging further research and application of CNFs in the field of active food packaging.

## 1. Introduction

As human society evolves and lifestyles change, the conveniences of modern civilization and high-tech living are increasingly accompanied by serious environmental problems such as global warming [1]. The reliance on plastics for food packaging is a major contributor to this negative environmental impact. Plastics derived primarily from petroleum, such as polystyrene [2], polyvinyl chloride [3], polypropylene, and polyamide [4], are preferred for their low cost, robust mechanical strength, and excellent moisture resistance and gas barrier properties. According to research by Roland Geyer et al., an estimated 8.3 billion tons of plastics have been produced, with 79% accumulated in landfills or natural environments, thereby posing a substantial threat to human health and ecological systems [5]. Plastic food packaging, particularly those containing phthalates, bisphenol A (BPA), and similar chemical additives, has been shown to leach into food, particularly under conditions of heat, light, or contact with fatty foods. This migration of chemicals can pose significant health risks, including endocrine disruption [6], reproductive and developmental effects such as reduced fertility, altered sperm quality, early onset of puberty in both males and females [7], and neurological and cognitive impairments [8]. In addition, prolonged exposure to these substances has been associated with an increased risk of obesity and metabolic disorders [9], as well as carcinogenesis [10]. In conclusion, the presence of chemicals such as phthalates and BPA in plastic food packaging is a significant public health concern, particularly in the case of long-term or high exposure. Consequently, the development of renewable resources to replace conventional plastics has emerged as a research priority, driven by growing concerns about environmental pollution and increasing consumer demand for healthier food options [11,12]. Biodegradable food packaging films derived from biopolymers such as polysaccharides and proteins are gaining popularity as environmentally friendly alternatives [13]. However, their development remains experimental due to limitations in barrier and mechanical properties. A number of strategies have been employed to overcome these challenges, including physical and chemical modifications [14,15,16], blending with other biopolymers [17,18,19,20], and the incorporation of nanofillers [21,22,23]. The use of reinforcing materials has proved particularly effective, with around 40% of the food packaging industry using such additives to improve barrier and mechanical performance [24,25]. Notable nanofillers, including silver nanoparticles [26,27,28], montmorillonite [29,30,31], zinc oxide [32,33,34,35], and titanium oxide [36,37,38,39], are favored for their non-toxic and biodegradable properties, making them ideal candidates for advanced smart food packaging applications. Research has increasingly focused on organic nanofillers derived from natural materials, which have demonstrated significant functionality [40]. This has led to burgeoning interest in the study of organic nanofillers, including cellulose and chitin nanoparticles [41,42]. Comparative studies have shown that the incorporation of montmorillonite and cellulose nanoparticles into alginate biopolymer films enhances the properties of bionanocomposites [43]. In particular, cellulose nanoparticles, which are renewable and derived from natural sources, significantly improve the hydrophobicity and tensile strength of carbohydrate-based biopolymers, highlighting their potential in advancing biodegradable packaging solutions.

**Figure 1:**
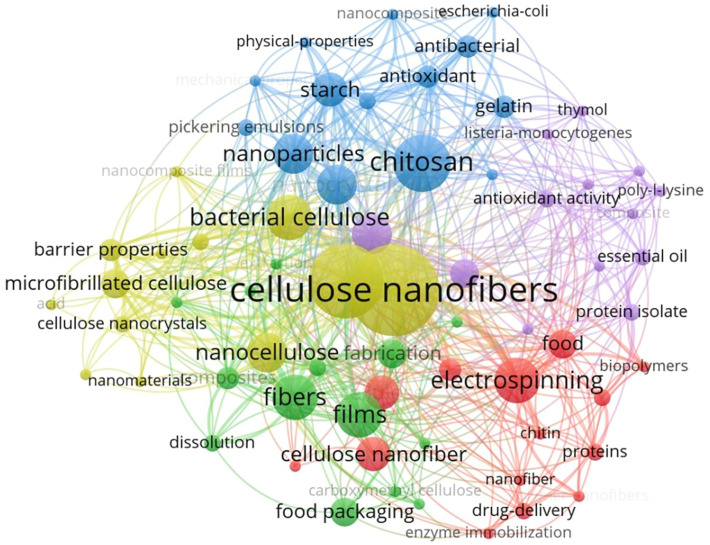
Clusters of cellulose nanofibers as keywords in publications on the manufacturing of food packaging (from 2021 to 2025).

Cellulose, with the chemical formula (C_6_H_10_O_5_) _n_ (where n ranges from 1000 to 30,000), is composed of glucose units linked by β-(1→4) glycosidic linkages and has a composition of 44.44% C, 49.39% O, and 6.17% H [44]. This biopolymer has attracted considerable attention in various applications, including drug delivery [45], food packaging [46], and functional foods [47]. Structurally, cellulose macromolecules originate from a D-glucopyranosyl group with a non-reducing C4-OH end and terminate in a reducing C1-OH group, which facilitates the formation of aldehyde groups upon ring opening. The polar and directional nature of the macromolecule is enhanced by the presence of three hydroxyl groups on each glucose unit: secondary hydroxyls at C2 and C3 and a primary hydroxyl at C6. These hydroxyl groups significantly influence the physicochemical properties of cellulose, contributing to its status as the world’s most abundant biopolymer, characterized by exceptional mechanical strength and economic viability. In particular, Turbak et al. pioneered the extraction of cellulose nanocellulose from wood in the 1980s, which subsequently led to the development of cellulose nanocrystals (CNCs) and cellulose nanofibers (CNFs) [48]. The development of nanotechnology has made it possible to produce cellulosic materials at the nanoscale, significantly expanding their potential applications. Among these, CNFs are increasingly being used as fillers in food packaging to improve the performance of active packaging materials. Due to their abundant hydroxyl groups, CNFs can be modified to improve compatibility with various materials, paving the way for additional high-value applications [49,50]. As a result, CNFs are used in the formulation of active food films, resulting in improved mechanical and barrier properties. Numerous studies have demonstrated the effectiveness of stabilizers in active food packaging, facilitating the controlled release of antimicrobial and antioxidant agents [51,52,53,54]. Furthermore, an examination of the Web of Science database for literature on CNFs from 2021 to 2025, using specific keywords, illustrates the evolution of research trends. The characteristics associated with these keywords are presented in Figure 1, providing a comprehensive overview. Although the existing literature has extensively reviewed the properties and applications of CNFs, most studies have focused on their functionalization in active food packaging. This paper systematically analyses and summarizes recent advances in CNF research, including modification techniques and application areas, providing valuable insights into the latest developments and guidance for optimizing production and commercial viability in active food packaging.

## 2. Sources of CNFs

The development of CNFs over the past few decades has been remarkable, positioning them as viable alternatives to petroleum-based feedstocks. This innovation facilitates the production of various commercial products that meet market demands while significantly reducing environmental concerns [55,56]. CNFs, which are derived from a variety of natural resources such as plant biomass, bacteria, and algae, have attracted considerable research interest due to their diverse application potential. CNFs are mainly derived from wood, agricultural residues, industrial by-products, bacterial biosynthesis, and aquatic organisms such as seaweed. Lignocellulosic materials mainly derived from plants serve as the primary sources of CNFs, including wood, cotton, jute, and flax. It is important to note that even when derived from identical raw materials, different parts of the plant can have different chemical compositions. Accordingly, CNFs can be classified according to their origin: straw fibers (e.g., corn, rice, wheat, and sorghum), bast fibers (e.g., flax, hemp, jute, sisal, and ramie), wood fibers (e.g., coniferous and broadleaf), grass fibers (e.g., bagasse, reed, and lobelia), bamboo fibers (e.g., moso bamboo, cedar bamboo, and white oleander), and seed fibers (e.g., copra fibers, cotton fibers, and kapok fibers) [57,58,59]. This classification underscores the diverse sources of nanofibers, each contributing unique properties and potential applications. The secondary sources of CNFs are mainly agricultural wastes, including animal manure, agricultural residues, and crop by-products such as rice husks and bagasse. In recent years, plant bio-waste has emerged as a significant category of bio-waste, contributing to approximately 5 billion tons of agricultural waste generated globally each year [60]. The cellulosic feedstock derived from these agricultural wastes shows promise as a lightweight aggregate and serves as a critical component in the production of bricks and insulation boards, offering significant potential for sustainable manufacturing practices [61]. The extraction of nanocellulose from agricultural wastes typically involves a two-step process. The first step entails the removal of lignin, hemicellulose, and other non-cellulosic substances, while the second step focuses on the extraction of nanocellulose from cellulose through either biological or physical methods. Enzymatic hydrolysis is the most commonly employed biological method. This process utilizes cellulase or a mixture of cellulases to catalyze the hydrolysis of cellulose, selectively degrading the amorphous regions while preserving the dense, crystalline regions. This results in the formation of nanocellulose with a defined length-to-diameter ratio [62]. The physical preparation method, which relies on mechanical treatments, utilizes high-energy inputs to disrupt the hydrogen bonding interactions between cellulose chains. This results in fiber fragmentation and fibrillation, producing nano-sized cellulose fibers [63]. Among the various physical treatment methods, high-pressure homogenization [64], microjet processing [65], and ultrasonication [66] are the most commonly used techniques for preparing cellulose nanofibers (CNFs). The third source of CNFs mainly comes from industrial processes, especially by-products from the food and beverage sector. Significant cellulose-rich bio-wastes include pressed bagasse, marc, and coffee grounds from brewing processes. In addition to CNFs from plants, CNFs can also be produced by various strains of acetic acid-producing bacteria, such as *Acetobacter*, *Gluconobacter*, and *Gluconacetobacter* [67]. The biosynthesis of these bacteria involves the conversion of monosaccharides into CNFs, which are then linked together to form a structured network. These bacteria are typically found in fermented foods, including vinegar and coconut juice, and have the ability to oxidize alcohols, aldehydes, sugars, or sugar alcohols to acetic acid in the presence of oxygen, facilitating the production of bacterial cellulose (BC) [68,69]. Compared with plant-derived CNFs, bacterial CNFs synthesized by microorganisms exhibit finer fiber diameters, superior mechanical properties, enhanced water-absorption capacity, and higher crystallinity, purity, and total surface area. However, a notable limitation of the bacterial synthesis of cellulose nanofibers is the time-consuming nature of the process, which poses challenges for large-scale industrial applications [70]. CNFs derived from marine biomass, particularly algae, are increasingly being considered promising future resources for nanocellulose isolation. The absence of lignin in marine algae, as opposed to terrestrial biomass, provides several advantages for nanocellulose extraction. The prevalence of pure CNFs and the absence of lignin in algae facilitate the production of high-quality CNFs, which are potentially applicable in a wider range of applications than those extracted from lignocellulosic biomass [71].

## 3. Characterization of CNFs

**Figure 2:**
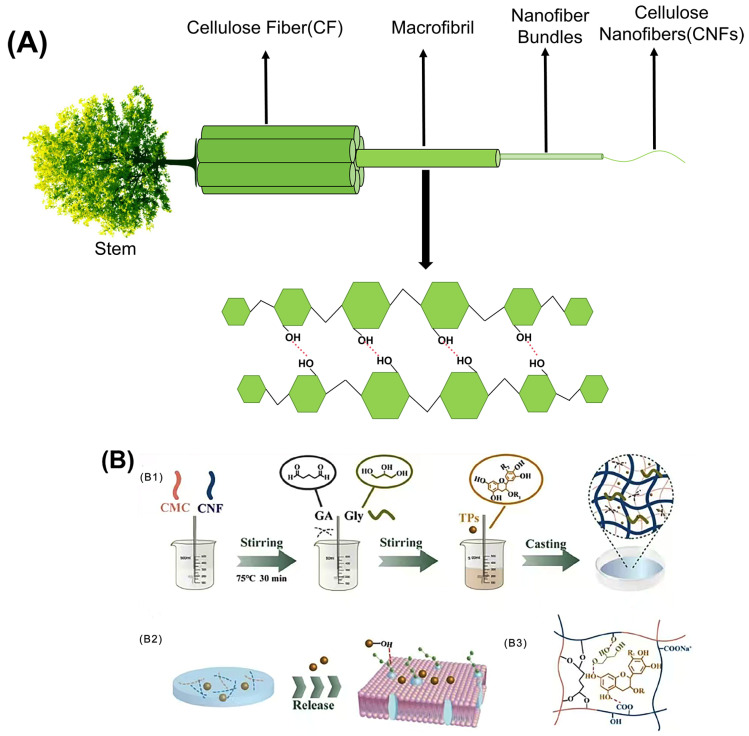
(**A**) Hierarchical structure of the wood-derived CNFs; (**B**) schematic of the preparation for the CNF composite films (**B1**) schematic representation of the preparation for the CNF composite films; (**B2**) molecular mechanism of tea polyphenols for food preservation; (**B3**) the interfacial bonding mechanisms between all components) [Reproduced from Ref. [69]].

Cellulose is fibrillated by intense mechanical processes, including milling, high-pressure homogenization, and microfluidization [72]. The resulting nanoscale cellulose is referred to as CNF (Figure 2A). CNFs are characterized by their crystalline and amorphous regions, exhibiting elongated filamentous structures with a length-to-diameter ratio typically greater than 50 and a diameter in the range of 5 to 30 nanometers, as defined by the Technical Association of the Pulp and Paper Industry (TAPPI). Compared with natural cellulose, CNFs exhibit improved properties, including high specific surface area [73], ease of surface modification [74], excellent mechanical strength [75], and favorable biosafety properties [76].

### 3.1. Mechanical Properties

Edible films used in food packaging must have robust mechanical properties to ensure the quality and integrity of food during transport, handling, storage, and distribution. The key parameters used to evaluate these mechanical properties include tensile strength (TS) and elongation at break (EB) [77]. Several factors influence the mechanical properties of CNFs, such as their source, preparation methods, crystallinity, porosity, and aspect ratio. No single parameter can be identified as the primary determinant of the mechanical properties. Among these, in terms of crystallinity, despite the lack of a highly ordered structure, CNFs exhibit a significant presence of cellulose crystalline regions, enabling them to exhibit excellent mechanical properties. Their strength has been reported to be 3–15 times greater than that of conventional cellulose composites [78]. The incorporation of CNFs into various polymer systems significantly increases the structural strength [79,80]. Ge et al. demonstrated this by combining CNFs with carboxymethyl cellulose, resulting in a composite film with remarkable tensile strength (~112.60 MPa) and favorable ductility (4.12%) [81]. Figure 2B shows a schematic of the preparation process of the CNF composite films. The extensive network formed by numerous CNFs with high aspect ratios facilitates a wider stress distribution under loading conditions, thereby improving both the mechanical strength and surface roughness of the films. Most studies investigating the effect of CNF incorporation on the mechanical properties of films report a significant increase in the tensile strength (TS) with the addition of CNFs. When blended with polymers such as proteins or chitosan, CNFs use their high aspect ratio, large surface area, and abundance of -OH to interact with polymer chains and act as cross-linking agents. This interaction promotes the formation of additional hydrogen bonds within the matrix, thereby increasing the TS. Guar gum-based films incorporating CNFs have been developed, resulting in a remarkable 885% increase in TS compared to control films [82]. However, it is important to note that at higher CNF concentrations, a decrease in TS was observed due to aggregation and the formation of weak points within the film matrix (the phenomenon of decreased TS increase effect caused by high concentration of CNFs). In addition, the elongation was negatively affected, and the increased stiffness of the films can be linked to the incorporation of CNFs and their effective dispersion throughout the matrix. A similar phenomenon was observed when CNFs were incorporated into collagen-based films, which showed TS up to 124 MPa at 10 wt% CNFs. CNFs have a higher density of hydrogen bonds than materials such as cellulose nanocrystals (CNCs). The presence of exposed CNFs on the fracture surface indicated that entanglement contributed to the increased modulus and toughness of the collagen-based composites. However, an excessive CNF concentration can lead to partial agglomeration. According to Griffith’s theory, the introduction of pre-existing cracks can increase stress concentration. The occurrence of these agglomerates is likely to result in larger, more macroscopic pre-cracks, further increasing the likelihood of stress concentration and subsequent material degradation [83]. CNFs significantly improve the mechanical properties of composite films by interacting with various substances. For example, when CNFs and graphene oxide (GO) were incorporated into soybean isolate protein (SPI), remarkable improvements were observed, with the tensile strength and Young’s modulus of the composite films increasing by 469.21% and 397.58%, respectively. This improvement can be attributed to the synergistic effects of physical entanglement, non-covalent interactions, and chemical cross-linking. A continuous network is formed by hydrogen bonding between GO, CNFs, and SPI, where the cross-linker interacts with the active groups and nanoparticles on the SPI surface to form a dense cross-linked network. This intricate structure significantly improves both the mechanical performance and overall integrity of the composite film [84]. In summary, the enhancement of TS in films by the incorporation of CNFs as fillers has been widely acknowledged. CNFs effectively fill the gaps between biomolecules such as polysaccharides and proteins, improving interfacial adhesion and facilitating efficient stress transfer. The degree of improvement was positively correlated with the CNF content, typically reaching an optimum concentration of around 10 wt% in the film matrix. Beyond this optimum threshold, the linear structure of CNFs tends to exhibit poor dispersion within the matrix, weakening hydrogen bonding interactions and reducing the improvement in mechanical properties. Determining the balance between the mechanical, optical, and barrier properties of CNF-based materials is critical. Any modification that disrupts the strong hydrogen bonds between the fibers may adversely affect the mechanical performance. Conversely, the ability to modify the density of hydrogen bonding allows the mechanical properties of CNF papers to be tailored to specific applications. For example, the availability of -OH in the system reflects a high degree of hydrogen bonding, which directly affects the stiffness of the CNF paper [85].

### 3.2. Emulsifying Property

The size of cellulose with carboxyl substitution and surface acetylation of cellulose nanomaterials significantly influences their emulsifying properties. In the context of Pickering emulsions, BC shows minimal emulsification ability, whereas semi-flexible CNFs and rigid CNCs effectively stabilize the emulsions. A study of CNF-based walnut oil gels showed that varying the CNF diameter—ranging from 5 to 10 nm, 10 to 20 nm, and 20 to 60 nm—had a pronounced effect on the properties. Oleogels with smaller CNF diameters showed higher oil-binding capacity (OBC, approximately 85%) and improved thermal stability. In addition, oleogel containing CNFs with diameters of less than 20 nm exhibits superior mechanical strength because of its compact structure. The protofibrillation process during CNF preparation increases the exposure of surface -OH, which serves as accessible hydrogen-bonding sites, thereby enhancing the interactions between CNFs and oil molecules during the oleogelation process. In the CNF/dihydromyricetin lotion system, CNFs interact with dihydromyricetin through hydrogen bonding. The concentration of CNFs in the mixture showed a remarkable synergistic effect on emulsion formation and its properties, suggesting a promising strategy for improving the shelf life of cellulose-based emulsions. Furthermore, the surface charge density and -COO- content of CNFs significantly influence their emulsification capacity and the stability of Pickering emulsions. Carboxymethylated CNFs were extracted from a bleached sulfur slurry via an etherification reaction followed by high-pressure homogenization. The surface charge density and size of the CNFs could be effectively tuned by varying the NaCl concentration and the number of homogenization cycles. The optimized CNFs exhibited high zeta potential (−71.2 mV) and suitable carboxylate content (1.81 mmol/g), which enabled them to irreversibly adsorb onto the surface of hydrophobic paraffin (PW) droplets. This adsorption formed an interfacial spatial barrier that generated substantial electrostatic repulsion between the PW droplets and prevented agglomeration. The CNF-stabilized PW emulsions showed remarkable stability, maintaining their integrity for up to six months or longer [86]. CNFs can be effectively combined with antioxidant essential oils to improve both the mechanical and antioxidant properties of films. CNFs were incorporated with cinnamon essential oil (CEO) into hydroxypropyl methyl cellulose (HPMC) films, resulting in active, printable, and heat-sealed materials. The TS of the CNF-enhanced HPMC films ranged from 22.91 to 25.23 MPa, comparable to those of commonly used food packaging materials such as high-density polyethylene (HDPE; 22–23 MPa) and low-density polyethylene (LDPE; 19–44 MPa). The extended-release time of CEO, together with improved oxidation resistance, sealability, and printability, makes these films particularly suitable for single-use plastic packaging applications, especially for high-fat foods [87].

### 3.3. Barrier Properties

The barrier properties of packaging films are critical for determining the shelf life of food products. Common permeability analyses focus on water vapor and oxygen. In food packaging, elevated water vapor and oxygen levels are associated with bacterial growth and food oxidation, respectively. Too high or too low barrier properties can compromise food quality and pose safety hazards. Gas permeation occurs via various mechanisms, with solution diffusion being the most common mechanism in film systems [88]. Permeation occurs through several key steps: first, gas diffusion to the film surface is driven by a concentration gradient, where gas molecules migrate from areas of higher concentration to those of lower concentration. Next, the gas molecules diffuse through the membrane and then desorb on the opposite side. The diffusion process is considered complete when the concentration of dissolved gas molecules within the composite film reaches saturation, and the concentration gradient across the film decreases. Several factors influence the gas permeability of films, including the preparation method, cellulose source, crystallinity, and environmental conditions. In particular, a porous arrangement of biomolecules typically has a higher gas permeability than a dense one. Although increased crystallinity improves the barrier properties of the film, it can also reduce its mechanical strength. Hydrogen bonding between CNFs plays a key role in determining the barrier properties. When CNFs are used as reinforcing agents, the improvement of the barrier properties of composite films is influenced by the degree of surface modification. A major challenge in the use of CNFs as alternatives to petroleum-based plastics is their inferior water vapor barrier properties compared to conventional materials. Research conducted by Hans Estrella Cainglet et al. involved the preparation of over 200 CNF films by casting and jet deposition, resulting in films with different basis weights, thicknesses, morphologies, porosities, and pore connectivity. Tests showed that pure CNF films could not achieve CC barrier properties comparable to those of synthetic plastic films, mainly due to the persistent voids and adsorption–desorption moisture diffusion mechanisms inherent in the films [89]. The phenomenon of double diffusion in pure CNF films is an inevitable consequence of both the film formation process and the intrinsic properties of CNFs. Protofibre size reduction and chemical modification are effective strategies for improving the water vapor permeability (WVP) of CNFs. The incorporation of tailor-made fillers that interact with the -OH on the raw CNFs can simultaneously reduce the microporosity and hydrophilicity, thereby improving the WVP of CNF films. These improvements position them as viable alternatives to synthetic plastics in food packaging and other applications. Jon Trifol and Rosana Moriana carried out pioneering research on different grades of nanofibers, in particular, CNFs with less than 1% lignin and lignocellulose nanofibers (LCNFs) with 16% lignin. Their results showed that the incorporation of LCNFs resulted in a 16% reduction in WVP and a 53% reduction in oxygen permeability. In addition, the presence of LCNFs improved the interfacial adhesion between the CNFs and facilitated the formation of more tortuous pathways for gas molecules, further contributing to the overall barrier performance [90]. CNFs exhibit an exceptionally low oxygen permeability of approximately 10^−23^ m^3^ m^−2^ s^−1^ Pa^−1^ in dry conditions, outperforming conventional petroleum-based film materials such as PET (polyethylene terephthalate) and EVOH (ethylene vinyl alcohol), which have oxygen permeabilities of 1 × 10^−19^ m^3^ m^−2^ s^−1^ Pa^−1^ and 6 × 10 ^−21^ m^3^ m^−2^ s^−1^ Pa^−1^, respectively. This superior barrier performance is primarily attributed to the highly crystalline structure, high polarity, and high aspect ratio of nanocellulose, which effectively mitigates food oxidation. The oxygen transmission rate (OTR) values of packaging films with CNFs chemically modified with succinic anhydride showed a significant decrease as the diameter of the CNF decreased. These results indicate that in the absence of substantial changes in CNF crystallinity, the oxygen barrier properties of CNF films are primarily influenced by their apparent density relative to nanofiber size, with smaller CNFs contributing to a denser structure that improves barrier performance [91,92]. By incorporating a functional polymer with a higher OTR of 38.5 cc/m^2^ day, the OTR of the CNFS films, initially measured at 2.56 cc/m^2^ day, was significantly reduced to 0.28 cc/m^2^ day. This reduction indicates that improved oxygen barrier properties occur primarily at the SCNF-FP interface. The synergistic improvement in the oxygen barrier can be attributed to the formation of additional interfacial hydrogen bonding between the FP and SCNFs, contributing to a denser or more interlocked interface [93].

### 3.4. Ultraviolet-Blocking Ability

Ultraviolet (UV) light lies between X-rays and visible light in the electromagnetic spectrum and is typically divided into three types: UV-A (400–320 nm), UV-B (320–280 nm), and UV-C (280–200 nm). UV radiation can directly induce DNA strand breaks, promote lipid peroxidation, facilitate protein carboxylation, and so on. Improving the UV resistance of food packaging is essential for minimizing photooxidation in packaged foods [94]. Consequently, improving the UV-blocking properties of packaging films has become a major area of research interest among scientists. Preservation of red lettuce in UV-blocking films has been shown to significantly increase the levels of total phenolics, anthocyanins, lignans, quercetin, and other bioactive compounds compared to red lettuce preserved in UV-transparent films [95]. As natural organic fillers, CNFs have minimal UV-blocking properties [96]. However, UV resistance can be improved by complexation with other polysaccharides, and the porous structure and fibrous surface of cellulose allow tunable light-scattering properties for optical tailoring [97]. Jung-Chan Kim et al. developed tempo-oxidized cellulose nanofiber (TOCNF) films with hydrophilic lignin, which exhibited remarkable UV protection with a blocking capacity of 99.9%, surpassing that of CNF–lignin composite films reported in other studies [98]. In addition, the use of whey protein concentrates and bacterial cellulose nanowhiskers (BCNW) in the formulation of edible films has demonstrated high light scattering properties, resulting in effective UV blocking (280 nm) [99]. The BCNW particles exhibited significant light-scattering properties, effectively blocking UV rays. In a separate investigation, antimicrobial composite films were synthesized using CNFs, lignin, and polybutylene adipate terephthalate (PBAT) as raw materials via a solution-casting method, achieving an impressive UV-blocking efficiency of over 95% [100]. UV blockers were also incorporated into the film and coating matrices. Wang et al. developed a multifunctional composite film by introducing micron-sized cellulose fibers (CMFs) and lignin–silver nanoparticles (Lig-Ag NPs) into the cellulose nanofiber (CNF) film network (CNF/CMF/Lig-Ag). This innovative composite has an interwoven microfiber and nanofiber structure that enhances its mechanical properties while providing antioxidant, antimicrobial, and UV-shielding properties. The composite demonstrated remarkable UV-shielding abilities of 98.2% for UVA and 99.4% for UVB, indicating its significant potential for UV protection applications [101]. Flavonoids, including quercetin, salicin, apigenin, and lignans, significantly enhance the UV-blocking properties of films. The effectiveness of these barrier properties is influenced by the number and positioning of -OH within the flavonoid chemical structures [102]. In addition, anthocyanins, recognized for their potent antioxidant properties, can absorb UV light due to their aromatic rings and flavonoid structures [103]. Curcumin-grafted rhythmic (2,2,6,6-tetramethylglycolan-1-yloxy) oxidized CNFs (CGTOCNFs) also exhibit strong UV-blocking properties due to their benzene rings and double-bonded moieties, as demonstrated in studies on chitosan films [104].

### 3.5. Biosafety

Natural cellulose and its derivatives are widely used in food processing because of their emulsifying and thickening properties and have been approved by the US Food and Drug Administration (FDA). Current research on the safety of CNFs has evolved from a focus on gastrointestinal motility to the study of various aspects of absorption, in vitro toxicity, and effects on nutrient assimilation. However, this research is still in its infancy and is largely dependent on in vitro cell experiments. Most studies, based on in vitro cell culture experiments, in vivo animal model assessments, and limited short-term clinical trials, have indicated the biosafety of CNFs [105]. In addition, CNF-based biomaterials, such as hydrogels and active films, have significant potential for application in both the biomedical and food industries. The surface chemistry and size of cellulose nanoparticles, together with the route and dose of exposure, may pose potential toxicity risks related to cellular uptake and tissue accumulation. Environmental factors such as pH, conductivity, and osmotic pressure can induce transient changes in the surface properties of CNFs. Alexandrescu et al. showed that wood-derived CNFs did not induce acute toxicity in fibroblast cell lines (3T3 cells), indicating their potential as a matrix regenerative medicine and wound healing [106]. Similarly, Shivaji-Biranje et al. reported that biofouling CNFs with casein resulted in TCNF–casein complexes that exhibited enhanced platelet adsorption and promoted targeted cell proliferation for accelerated and controlled wound healing [107]. Overall, the current literature suggests that CNFs are generally non-cytotoxic with negligible gastrointestinal absorption. However, further studies are essential to establish a definitive safety profile [108,109,110].

## 4. Functionalization of CNFs

**Figure 3:**
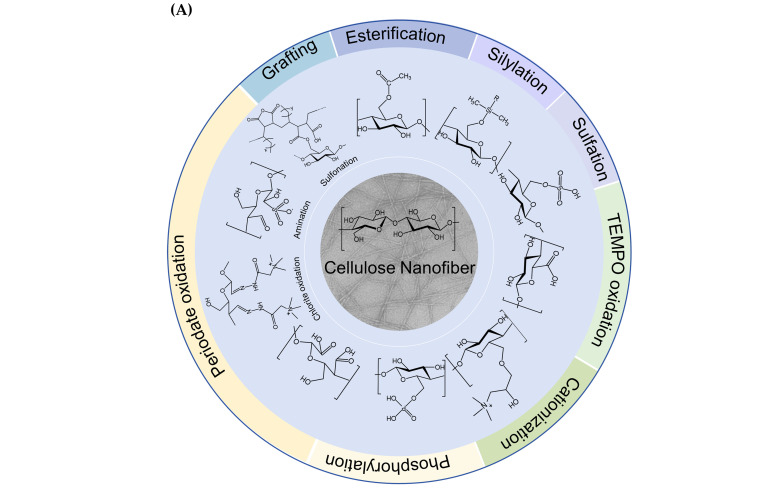

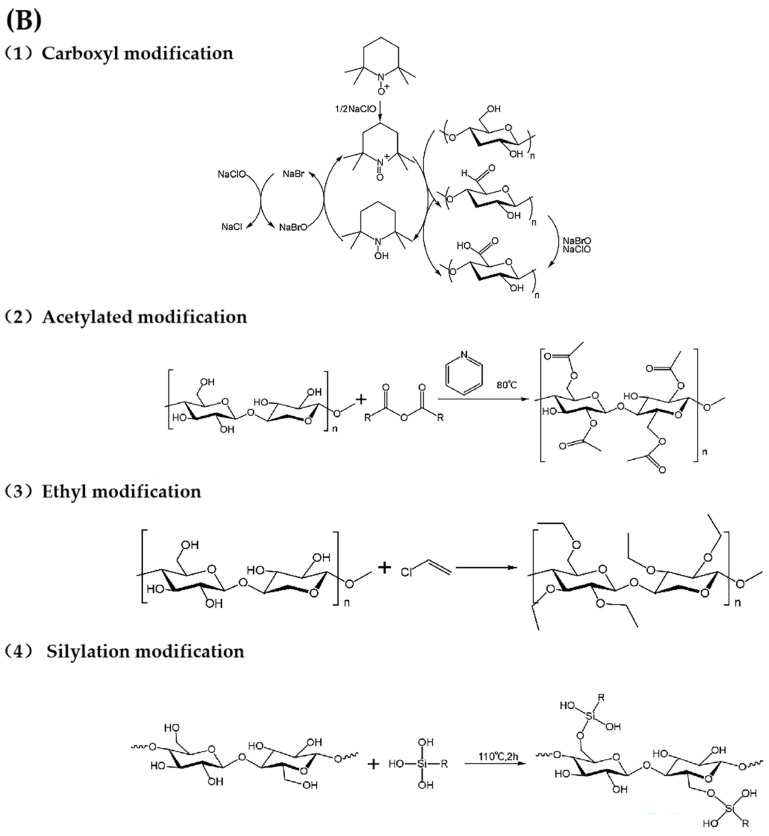
(**A**) Chemical functionalization of CNFs [116], (**B**) chemical reactions of CNFs functionalization process.

In response to the growing demand for sustainable products, there has been a significant increase in research interest in CNFs owing to their renewability, biodegradability, and exceptional strength. Advances in nanotechnology have made it possible to precisely process CNFs at the nanoscale using various techniques, including high-pressure homogenization, ball milling, and high-intensity ultrasound treatment. However, challenges such as rigid energy requirements and high hydrophilicity limit their wider application. Functionalization of CNFs can significantly improve their performance and extend their utility. Natural CNFs have a high density of -OH, mainly located at the C2, C3, and C6 positions, and they are highly reactive and facilitate the conversion of these groups into various surface functional groups. This conversion is achieved by chemical modification methods that introduce specific functional groups at different hydroxyl positions in the D-glucose units, allowing precise control of their content and distribution. In addition, chemical or biological pre-treatment prior to mechanical processing can improve preparation efficiency and reduce energy consumption. Currently, many chemical modification techniques based on -OH have been developed [111], including oxidation [112], esterification [113], and sulfonation [114] (Figure 3). The reactivity of -OH in cellulose varies at different positions depending on the reaction type. In general, reversible reactions mainly affect C6-OH, whereas irreversible reactions are more likely to occur at the C2-OH. Consequently, during esterification, C6-OH exhibited the highest reactivity, whereas during etherification, C2-OH was the most reactive. Furthermore, the induction effect of neighboring substituents influences the acidity and dissociation of -OH, resulting in the following order: C2 hydroxyl > C3 hydroxyl > C6 hydroxyl. This hierarchy suggests that secondary -OH are primarily reactive in alkaline environments, whereas primary -OH are more reactive in acidic conditions [115]. These findings provide a strategic framework for selecting appropriate chemical modification methods to target -OH at specific sites.

### 4.1. Carboxyl Modification

The introduction of -COOH onto the surface of CNFs is primarily achieved via oxidation reactions. The oxidation of CNFs can be divided into selective oxidation and non-selective oxidation processes, the former targeting specific hydroxyl groups at specific positions and the latter involving indiscriminate oxidation [117]. Common reagents for non-selective oxidation include sodium hypochlorite, persulfate, and hydrogen peroxide, but these can cause significant CNF degradation, making it difficult to control the extent of fiber oxidation and degradation. Selective oxidation of secondary-OH is usually achieved using periodate or periodate salts [118], whereas selective oxidation of primary-OH can be achieved by various methods, including nitric oxide oxidation, sodium chlorate/sodium chlorite/sodium bromate oxidation, nitrite/phosphoric acid systems, hypochlorite oxidation, and TEMPO oxidation. These techniques enable more controlled modification of CNFs to improve their functionality [119]. Among the oxidation methods, TEMPO (2,2,6,6-tetramethylpiperidine-1-oxide radical) oxidation is the most widely used because of its advantageous selectivity, mild reaction conditions, and high efficiency. TEMPO-mediated oxidation typically occurs in the presence of sodium hypochlorite (NaClO) and sodium bromide (NaBr) and selectively converts the C6-OH of CNFs to -COOH, thereby enhancing the properties of CNFs. The production of CNFs from sources such as corncob bagasse, waste wood, and bacterial cellulose has been demonstrated, with yields ranging from 25% to 34%. In particular, bacterial cellulose and corn cobs exhibited superior transparency, crystallinity, and oxidation efficiency compared to other sources, providing valuable insights for exploring the potential applications of CNFs in various fields. The TEMPO-mediated oxidation process may inadvertently generate small aldehyde groups. To minimize aldehyde formation while ensuring selectivity for primary hydroxyl groups, the reaction must be maintained at pH between 9 and 11 using NaClO as the primary oxidant and TEMPO and NaBr as catalysts. However, current methods for producing carboxylated CNFs often require harsh reaction conditions that compromise the high aspect ratio of the products, resulting in low yields, significant environmental impacts, and reduced utility. An innovative method for preparing carboxylated CNFs was developed, which is comparable to the classical TEMPO method [120]. This new method uses a variety of low-melting-point solvent feedstocks and is characterized by ease of synthesis, cost-effectiveness, non-pollution, and biodegradability. Using citric acid, choline chloride, and water as starting materials, this method effectively breaks the hydrogen bonds between cellulose macromolecular chains to facilitate -COOH functionalization. This process is further enhanced by the use of a high-speed stirrer, high-frequency ultrasonic reactor, or high-pressure homogenizer to produce nanofibers. The resulting CNFs achieve yields of up to 90.12%, consisting predominantly of single precursor fibers with diameters of approximately 3 nm and lengths exceeding 10 µm, resulting in impressive aspect ratios of up to 2500 and -COOH contents of 1.5 mmol/g. In addition, the exceptional stability of CNF suspensions, even at elevated concentrations, enhances their storage, transport, processing, and utilization.

### 4.2. Acetylated Modification

The acetylation of CNFs is one of the most extensively studied esterification reactions and serves as a simple method to reduce the density of -OH on nanocellulose. The basic mechanism of acetylation involves the partial substitution of hydroxyl -OH groups on CNFs with acetyl groups. The initial stage of acetylation primarily targets the most accessible -OH, typically on the surface or within disordered/amorphous regions of CNFs. Once the surface -OH are fully substituted, the reaction proceeds to less accessible -OH, such as those within the crystalline regions of CNFs [121]. Due to hydrogen bonding interactions within and between CNF molecules, acetylation facilitates the solubilization and activation of CNFs, thereby weakening the intermolecular forces between the CNF macromolecules and increasing the homogeneity and reactivity of the material. Acetylation is quantified by the degree of substitution (DS), defined as the average number of -OH substitutes per dehydrated glucose unit. Theoretically, the maximum DS achievable is 3.0, depending on the specific reaction conditions [122]. CNFs with a DS of approximately 1 tend to exhibit better dispersion in polar solvents, whereas a DS closer to 3.0 favors dispersion in hydrophobic nonpolar solvents. A common multiphase reaction for acetylation reaction involves the use of a CH_3_COOH/(CH_3_CO)_2_O system with glacial acetic acid and acetic anhydride as reactants. Acetylated CNFs and synthesized degradable composite films were prepared using pinewood, demonstrating the significant potential of composite films as environmentally friendly materials for packaging applications [123]. In addition to glacial acetic acid and acetic anhydride, CNFs have also been modified with citric acid, resulting in adsorbents that effectively remove heavy metals from water [124]. In addition, maleic anhydride has been grafted in situ onto CNFs to create adsorptive films with remarkable adsorption capacities (up to 1620 mg/g within 12 h), meeting the growing demand for rapid and efficient adsorption of positively charged proteins [125].

### 4.3. Ethyl Modification

Ethyl CNFs are partially ethylated CNFs ether produced by the reaction of alkali cellulose with ethyl chloride, typically using a NaOH/C_2_H_5_Cl system. The conditions for ethylation are much more stringent than those for acetylation, requiring higher NaOH alkalinity, higher reaction temperatures, and longer reaction times. Under alkaline conditions, the ethoxyl groups first replace -OH at the C2 position, followed by substitution at the C6 and C3 positions. The relatively slow reaction rate helped to control the fibrous morphology of the CNFs, preventing complete etherification and subsequent conversion into powder. Different types of EC are characterized by their viscosity, molecular weight, and degree of substitution (DS). N-type EC, which is commonly used in commercial applications, is produced by precisely controlling the substitution of -OH on the surface. The reaction is typically complete when the ethoxyl content reaches 47.5–49%, corresponding to a DS of approximately 2.5. Pure ethyl CNFs exhibit high mechanical strength, flexibility, heat resistance, and insolubility in water while remaining soluble in various organic solvents, including ethanol, methanol, resins, and oils. In particular, ethyl CNFs became highly insoluble when the DS exceeded 2.8 [126]. Zhou et al. successfully recovered CNFs from rice straw and ethylated and modified them to produce ethyl CNFs for the preparation of biodegradable films. The yield of ethyl CNFs reached 79.60%, and the DS ranged from 2.0 to 2.5. The resulting films, at a concentration of 0.04 g/mL, exhibited a smooth surface, biodegradability in soil, and tensile strength of up to 44.60 MPa, providing an environmentally friendly and sustainable alternative to conventional plastic films [127].

### 4.4. Silylation Modification

Silanization modification involves the hydrolysis of alkoxy groups in silane coupling agents to form silanol, which can then react with -OH on the surface of CNFs to form stable covalent bonds. The substitution of -OH with silanol functional groups significantly affects the hydrophobicity of CNFs. Various silane derivatives, such as N-(β-aminoethyl)-γ-aminopropyltrimethoxysilane (AEAPTMS) and 3-aminopropyltriethoxysilane, have been used to modify CNFs, with degree of substitution (DS) values typically ranging from 0.6 to 0.9. At higher silylation levels (DS > 0.9), the structural integrity of the crystals can be compromised, resulting in the loss of the original CNF morphology. Therefore, a careful balance between the degree of silylation and the preservation of the cellulose morphology is essential during the silylation process [128]. The use of appropriate silylation reagents with shorter reaction times can help maintain the integrity of CNF morphology. Peresin et al. investigated two well-established chemical functionalization methods using hexamethyldisilazane (HMDS) and (3-aminopropyl) trimethoxysilane (APTES) to produce hydrophobic films that effectively hinder water–CNF interactions. In addition, the silylated films exhibited a reduced affinity for oxygen. In comparison, HMDS-modified CNF films showed a greater reduction in surface hydrophilicity than APTES-modified films, even at lower surface substitution rates, thereby improving their oxygen permeability properties [129]. Silanization modification can also enhance the antimicrobial properties of CNFs. Although CNFs are not inherently antimicrobial, they can be functionalized with quaternary ammonium [130], silane [131], and other agents to improve their antimicrobial efficacy. The salinization of CNFs with 3-aminopropyltrimethoxysilane (APMS) resulted in a significant reduction in bacterial concentrations. In particular, *Bacillus subtilis* was reduced by 1.3 log, *Staphylococcus aureus* by 1.8 log, and *Escherichia coli* by 3.8 log, demonstrating the effective inhibition of microbial proliferation on the treated polymer surface [132]. The research team also reported the development of contact-active antimicrobial materials by functionalizing CNF films with three different aminosilanes (3-aminopropyltrimethoxysilane (APMS), 2-aminoethyl-3-aminopropylmethoxysilane (DAMS), and 3-(2-(2-aminoethylamino) ethylamino), propyltrimethoxysilane (TAMS)). In particular, very low concentrations of TAMS completely inhibited the growth of Gram-positive bacteria within 24 h. The authors found a correlation between the length of the aminoalkyl chain and the associated antibacterial activity, highlighting the potential for tailoring antimicrobial properties through chemical modification [133].

### 4.5. Physical Modification

Cold plasma technology is a non-thermal treatment method used to modify and preserve food. Various electrical methods, including pulsed discharges, microwaves, ultrasound, and corona discharge, can generate cold plasma in gaseous environments. This process produces a range of reactive species, such as charged particles, quasi-ultraviolet photons, free radicals, and other reactive substances (e.g., hydrogen, nitrogen, and oxygen) that effectively inactivate harmful microorganisms on packaging materials and food products [134]. Cold plasma is used extensively in food technology, particularly for pesticide residue removal, food purification, toxins degradation, enzyme inactivation, and material modification. Its potential is particularly great in improving the properties of CNF films. Cold plasma treatment can improve film properties by reducing oxygen permeability, increasing mechanical strength, providing excellent barriers to oils and fats, reducing breathability, and improving antibacterial properties. Cold plasma-treated CNF films have been shown to effectively inhibit microbial growth in dairy, meat, and seafood products and to retard oxidative browning in fruit and vegetables. These films are increasingly being used in the development of aseptic packaging for meat, fruit, and vegetables. However, the inherent high wettability of CNF-based films often limits their performance as an efficient alternative in the packaging industry. To address this challenge, Ana Oberlintner et al. used fluorocarbon plasma treatment to increase the hydrophobicity of CNF surfaces. The nanofibril films were exposed to CF4 plasma, achieving a rapid hydrophilic-to-hydrophobic conversion in less than 10 s. After only 30 s of plasma treatment, a water contact angle saturation of approximately 130 ± 5° was achieved, demonstrating the effectiveness of CF4 plasma treatment for the ultrafast transformation of CNF surfaces from hydrophilic to hydrophobic [135].

Ultraviolet (UV) radiation is known to cause the cleavage of chemical bonds within CNFs’ molecular chains, facilitating the formation of carbonyl groups on the fiber surface and increasing the polarity of the fiber. This modification technique is fast and efficient and can be precisely controlled. However, it is associated with significant energy consumption, especially in large-scale production. Therefore, a careful evaluation of the economic benefits of using UV radiation is essential, considering both energy costs and environmental impact. High-energy radiation sources such as alpha and beta particles, as well as X-rays and gamma rays, have been used in various industries, including nuclear power, healthcare, and aerospace [136]. Currently, radiation techniques such as cross-linking, radiation-induced polymerization, and polymer degradation are used for polymer modification. Radiation technology affects polymers mainly through two main aspects: (1) its versatility in processing polymers of different shapes and sizes and (2) the prevalence of cross-linking reactions during irradiation. Gamma irradiation can induce both physical and chemical changes, including cross-linking, decomposition, and unsaturation, thereby increasing the strength and stiffness of polymer materials. The application of specific doses of radiation can improve the mechanical properties by generating free radicals within the composite material. When low-energy radiation interacts with materials, the photoelectric effect can transfer energy to electrons, causing them to be ejected from the atoms. In natural fibers, gamma radiation typically generates free radicals that facilitate further cross-linking, including initiation, propagation, and termination reactions. Tarik Hossain synthesized CNFs from waste jute sacks and used them as reinforcing agents in unsaturated resin-based composites. The application of high-energy gamma radiation doses (2.5, 5.0, and 7.5 kGy) throughout the manufacturing process resulted in significant performance improvements. In particular, with the incorporation of 0.2% CNFs and a gamma dose of 5.0 kGy, improvements in mechanical properties were observed, including a 210% increase in tensile strength, a 200% increase in flexural strength, a 12.5% reduction in elongation at break, and a 56% increase in impact strength. This study highlights the effectiveness of CNFs as a reinforcing agent in composite materials, facilitated by the application of targeted doses of gamma radiation to enhance their mechanical properties used as reinforcing agents [137].

## 5. CNF-Based Films Containing Biopolymers, Functional Materials, and Their Combinations

Despite extensive research into CNF films as a sustainable alternative to synthetic plastics, their commercial applications have remained limited. Key challenges include the poor water vapor barrier properties, suboptimal film formation, and high brittleness of CNF films, which hinder their effectiveness in environmentally friendly food packaging applications. To address these limitations, various biopolymers and nanomaterials were incorporated to improve performance, as shown in Table 1 and Table 2. Biopolymers such as chitosan [138], carboxymethylcellulose (CMC) [139], starch [140], and proteins [141] have been used to improve the functionality of CNF films. The incorporation of these biopolymers is an effective strategy to reduce costs, improve biodegradability, and enhance overall performance. The incorporation of functional nanomaterials into CNF films enhances their antimicrobial properties and improves their gas and water barrier properties. Examples include graphene oxide (GO) [142], ZnO nanoparticles (ZnONPs) [143], and lignin nanoparticles [144]. In addition, bioactive compounds such as grapefruit seed extracts, curcumin [145], plant polyphenols, and extracts from mint and pomegranate peels [146] and essential oils such as savory oil [147] have been explored. The incorporation of these functional materials has facilitated the development of CNF films with antimicrobial, antioxidant, plasticizing, and UV-blocking properties. In addition, smart CNF films can be prepared by incorporating anthocyanins, which act as pH indicators and are derived from natural pigments. These films can detect the freshness and quality of packaged foods by observing color changes [148]. Recent review articles have focused on various aspects of CNFs and their composite films, including CNF-based hybrid aerogels, the mechanical properties of CNF papers and their bio-nanocomposites, and advances in the preparation of CNFs. This paper aims to review the use of different polymers, functional fillers, and their combinations to improve the performance of CNF-based films.

### 5.1. CNF-Based Films with Biopolymers

In the last decade, researchers have increasingly focused on improving the properties of films by blending CNFs with various biopolymers, as shown in Table 1. Among these biopolymers, chitosan, starch, and proteins are most commonly used in combination with CNFs.

Chitosan, a natural biodegradable polymer, has excellent film-forming properties, antimicrobial activity, UV protection, and low oxygen permeability. The compatibility of this reagent with CNFs is attributed to strong hydrogen bonding interactions. Qin studied the composite films formed by TEMPO-oxidized CNFs (TOCN) and chitosan and found that the negative charge of TOCN and the positive charge of chitosan enhance hydrogen bonding and electrostatic interactions. These improvements result in improved mechanical properties and excellent biocompatibility of the nanocomposite films [149]. In addition, functionalized CNFs can be blended with chitosan to form highly effective antimicrobial bioplastics. For example, cationic hyperbranched polyamide and lignosulphonate-functionalized CNFs form a supramolecular network with chitosan, resulting in tensile strength of up to 50.4 MPa, as well as excellent antimicrobial activity, water resistance, UV shielding, and thermal stability [150].

Starch is another commonly used biopolymer for biodegradable film production, but it faces challenges such as structural brittleness, poor long-term stability, low mechanical strength, high gas permeability, low heat distortion temperature, and reduced water resistance. These limitations hinder its use in food packaging. However, blending starch with CNFs promotes effective interfacial embedding through hydrogen bonding, which improves the mechanical strength, stiffness, and durability of the film while addressing permeability and solubility issues [151]. The high crystallinity and large surface area of CNFs facilitate strong interactions with the starch matrix, promoting uniform film dispersion [152]. Research has shown that starch composite films prepared with TEMPO-oxidized CNFs (TONF) and natural tapioca starch (NTS) exhibit significant improvements in mechanical properties and wet strength due to the synergistic effects of enhanced crystallinity and hemiacetal cross-linking, making them viable alternatives to disposable packaging films [153]. In addition, surface-modified CNFs are widely used in starch films. Modifications using urea/NaOH (UA), oxalic acid (OA), citric acid (CA), and (3-mercaptopropyl) trimethoxysilane (MT) have been shown to improve the tensile strength, tensile modulus, crystallinity, barrier properties, and hydrophobicity of the composite films. In particular, ball milling facilitates the physical integration of starch and CNFs, generating metal–organic supramolecular interactions between -OH groups and Ca^2+^ ions, resulting in new starch-based materials with high mechanical strength and transparency [154]. While an increase in CNF content typically improves mechanical properties, there is a critical threshold beyond which excessive fiber accumulation can interfere with the stress transfer mechanism. Achieving the optimum mechanical performance, therefore, requires careful consideration of the following factors: dispersion, fiber size, and loading, particularly in different composite applications. In addition, the inherent hydrophilicity of starch-based materials is a significant limitation. The introduction of CNFs reduces water permeability, making these composites more resistant to water absorption than pure starch. Research by Chen, Liu, and Chen showed that the incorporation of bamboo, cotton, and sisal CNFs into starch films resulted in a significant reduction in water vapor permeability (WVP), with decreases of 27.60%, 21.98%, and 18.91%, respectively [155]. This phenomenon is attributed to the high crystallinity of CNFs, which effectively extends the water-vapor diffusion path. Several other studies have confirmed that the addition of CNFs consistently reduces water vapor permeability [156,157,158]. In the case of modified starch, CNFs also exert beneficial effects. Soni et al. investigated the blending of three different starch types—hydroxypropyl starch (HPS), acetyl starch (AS), and acetyl oxidized starch (AOS)—with TEMPO-oxidized cellulose nanofibers (TOCNF). The TOCNF/modified starch films formulated at a ratio of 60% CNF and 40% starch (1:0.6 wt%) exhibited minimal swelling and satisfactory wet strength. TOCNF-reinforced starch films are poised to serve as the next generation of renewable, biodegradable, water-resistant, high-performance green packaging films [159].

Organic-based materials are increasingly being explored as sustainable alternatives to plastic packaging, promoting the use of environmentally friendly natural resources. These organic materials include not only polysaccharides but also proteins, which are recognized as excellent candidates for the production of food packaging films. Of particular note are collagen and gelatin, which are primarily derived from the bones, cartilage, and skin of fish and other animals. Gelatin is a promising biopolymer owing to its superior film-forming ability, biodegradability, abundance, and economics. However, proteins can present challenges in packaging applications, including high hydrophilicity, low mechanical strength, brittleness, and opacity. The incorporation of CNFs can address these shortcomings and improve the hydrophobicity, tensile strength, and plasticity of films.

Wang et al. investigated the blending of different forms of CNFs with gelatin to produce composite films for tomato preservation. The gelatin-based composite film containing 0.3% CNF exhibited the lowest water vapor permeability (WVP) of 1.97 × 10^−4^ barrer, low oxygen permeability (OP) of 2.54 × 10^−2^ barrer, high transparency (85.28%), and excellent mechanical properties (tensile strength, σ = 47.45 MPa; Young’s modulus, E = 1.84 GPa) [160]. In addition to animal proteins such as casein, whey, keratin, and egg white, plant proteins derived from wheat, maize, soybeans, and peas, as well as by-products from the food industry, provide affordable and concentrated protein sources. Soy protein isolate (SPI) is particularly valued for its nutritional value and functional properties, consisting of 7S (β-conglycinin) and 11S (glycinin) proteins [161]. The stability of the glycin-rich structure is attributed to hydrogen bonding, hydrophobic interactions, and disulfide bonds, which enhance film-forming and antioxidant properties. Gonzalez et al. prepared CNFs from low-cost agro-industrial by-products such as soybean hulls and pods and used them as nanoreinforcing agents for soy protein films. Their results showed that SPI-CNF films exhibit robust tensile strength and reduced water vapor permeability. The incorporation of CNFs also reduced the film’s susceptibility to water, with a significant reduction in total soluble matter and water swelling as the contact angle increased [162]. Qin et al. investigated the effects of different types of CNFs from the same biological source on the physicochemical and mechanical properties of protein films [163]. Different chemical pre-treatments and mechanical processing methods resulted in CNF protein films with the highest light transmittance. Variations in surface micromorphology, -OH density, and hydrogen bonding sites among different types of CNFs resulted in significant differences in the spatial distribution and intermolecular cross-linking within the protein matrix. In addition, modified CNFs are widely used in protein films. Zhang et al. used bis-formaldehyde carboxylated CNFs as a cross-linking agent that reacts covalently with proteins, resulting in composite films with reduced water content, swelling, water vapor permeability, and relative oxygen permeability [164].

### 5.2. CNF-Based Films with Functional Materials

CNF-based films are increasingly being enhanced with functional materials to improve their properties, as summarized in Table 2. These materials can be divided into two groups: (i) those that improve their physical and mechanical properties and (ii) those that introduce additional functionalities such as antimicrobial, antioxidant, UV-blocking, and sensing or tracking capabilities. Materials such as chitin nanowhiskers, graphene oxide (GO), and nanoclay have been used to enhance the mechanical, thermal, and barrier properties of CNF films. For example, chitin nanocrystals can be combined with TEMPO-oxidized CNFs to form self-assembled films through electrostatic interactions between the amino groups of chitins and the -COOH of CNFs. This process, facilitated by EDC/NHS-mediated chemical cross-linking, results in composite films with lower tensile strength than monolithic films but higher elongation, achieving almost 2.3 times the elongation of TOCN films [165]. In addition, the incorporation of functional agents such as curcumin, carbon dots (CDs), grapefruit seed extract, essential oils, and metal ions has been shown to improve the hydrophobicity and antimicrobial properties of CNF films. Specifically, the inclusion of CDs resulted in a 26.1% increase in tensile strength while providing remarkable UV-blocking capabilities (blocking 98.8% of UV-B and 87.4% of UV-A radiation) without compromising film transparency. CD-loaded CNF films also demonstrated strong antioxidant properties, achieving 98.2% and 78.8% removal of ABTS and DPPH radicals, respectively. In addition, these films exhibited effective antimicrobial activity, significantly reducing the growth of *E. coli* and *Listeria monocytogenes* after 12 h of exposure [166]. The use of CNFs as Pickering stabilizers for water-insoluble liquids, including essential oils and plant extracts, has attracted considerable interest in food packaging applications. This approach can inhibit lipid oxidation in meat products, thereby extending their shelf life and reducing the spread of foodborne pathogens associated with diseases such as listeriosis, cyclosporiasis, and salmonellosis [167]. Anionic CNFs have been used as stabilizers for thyme essential oil (TEO) in active food packaging. The adsorption of CNFs at the oil/water interface limits agglomeration and requires rheological stabilization to prevent the buoyancy of larger droplets (>10 μm). The thickening effect resulted in increased viscosity (exceeding 0.1 Pa S at 10 s^−1^) and yield stress of about 0.9 Pa. Dilute emulsions show excellent film-forming properties, whereas concentrated emulsions are effective for paper coating and have successfully inhibited the growth of both Gram-negative (*Escherichia coli*, *Salmonella typhimurium*) and Gram-positive (*Listeria monocytogenes*) bacteria [168]. Metal nanoparticles are increasingly being utilized as functional materials in CNF films. Sodium lignosulfonate (SL) and ZnO NPs serve as functional fillers and structural components, respectively, enhancing the films’ UV-blocking, antioxidant, and antimicrobial properties and providing good mechanical, thermal, and humidity resistance. Seafood products, recognized as a rich source of high-quality protein and polyunsaturated fatty acids, are particularly susceptible to deterioration under frozen storage conditions, underscoring the growing demand for smart materials in packaging. Anthocyanins, natural water-soluble colorimetric indicators belonging to the flavonoid phytochemical group, have been employed to develop pH-sensing films. Nanocomposite films incorporating CNFs and cellulose acetate have been prepared with natural anthocyanins for ammonia detection. The presence of CNFs enhances the surface uptake of anthocyanins, enabling the films to function as ammonia vapor sensors through a color change correlated with pH. Characterization of the films revealed a tensile strength of 29 MPa, thermal stability of up to 288 °C, and potential for conversion into heat-sealed bags [169]. There is growing consumer demand for natural ingredients as alternatives to chemical preservatives. Antimicrobial agents, including short-chain fatty acids, extracellular polysaccharides, vitamins, enzymes, bacteriocins, and trienoic acids, not only promote human and animal health but also serve as bio-preservatives for food. Among them, bacteriocins have been extensively used in active packaging. For example, Mapelli et al. developed an antimicrobial active packaging system aimed at reducing *Listeria monocytogenes* populations in ready-to-eat smoked salmon. In this system, a protein extract containing sakinomycin (produced by *Lactobacillus saccharomyces cerevisiae* Lb 706 from a low-cost medium of deproteinized cheese whey) was adsorbed onto CNFs, resulting in a reduction of Listeria populations via three logarithmic cycles [170]. In addition, Maresca and Mauriello developed an antimicrobial packaging film comprising *Streptomyces lactis* peptides within a CNF matrix, which demonstrated efficacy in vitro against the indicator microorganisms *Brochothrix thermophacta* and *Listeria innocua*. Following contact with hamburgers, a reduction in *Listeria* populations of approximately 1.3 log cycles was observed [171]. These studies demonstrate that CNF-based active packaging enhanced with functional substances significantly improves antimicrobial, UV resistance, and mechanical properties. With the increasing consumer demand for food freshness and environmental sustainability, the functional properties of materials used in preservation films are increasingly being prioritized. CNFs can be effectively used in food packaging by blending them with other biopolymers and incorporating nanomaterials to increase their strength and reduce gas and moisture permeability. Sustainably sourced CNF films with excellent mechanical and barrier properties, integrated antimicrobials, and smart features offer an environmentally friendly alternative to traditional plastics used in food packaging.

**Table 1:** Selected recent studies on CNF-based composite films with biopolymers.

Materials	Preparation	Key Finding	References
Chitosan/switchgrass-based lignin-containing CNFs	Physical blending: chitosan was dissolved in a 1.5 wt% glacial acetic acid solution, and the CS solutions were obtained by constant stirring at 50 °C for 3 h.	CS/LCNF maintained a ductility of 503% and showed a 46.7% higher tensile strength with a Tmax and WCA that were increased by 2.6 °C and 11.06°, respectively.	[172]
Poly (lactic acid)/nanosilver-decorated cellulose/chitosan/lignocellulose nanofiber	Physical blending: 1 g (dry matter) of each nanofiber was dispersed in 200 mL of 1 mM AgNO_3_ aqueous solution, followed by ultrasonication (40 kHz, 20 min). Then, the specific quantity of *L. salicaria* extract was added to the mixture at 60 °C and agitated by a magnetic stirrer for 30 min.	Better dispersion and compatibility of LCNF and CHNF, along with a high amount of the loaded AgNPs, resulted in strong interactions, smooth films, better-controlled release, surface hydrophobicity, enhanced barrier properties, and reinforcing impact.	[173]
CNFs/carboxymethyl chitosan	Physical blending: 14 wt% CMC solution, CNF dispersion (1%) was mixed with TA at different TA to CNF mass ratios under constant stirring for 30 min, and ultrasonic treatment was performed to remove the bubbles.	The developed anti-fog film had high mechanical strength and excellent UV shielding properties, as well as good antibacterial and antioxidant properties. The bilayer anti-fog film could effectively prevent the generation of fog, delay the Browning, inhibit mildew, improve the overall acceptability, and effectively extend the shelf life of white Hypsizygus marmoreus.	[174]
Sodium alginate/CNFs/ethyl cellulose/polyvinyl butyral	Physical blending: using a coating device, the produced SA/CNF solution was uniformly applied to the dried Ca^2+^ ion-filled base paper. The SA/CNF coated paper was dried in an oven at 65 °C for 40 min. The EC/PVB mixture was coated on the surface of SA/CNF oil-proof paper and dried for 30 min in an oven at 45 °C to create SA/CNF/EC/PVB water- and oil-proof paper.	The water- and oil-proof paper showed excellent water repellency (Cobb value: 1.1 g/m^2^), oil repellency (kit rating: 12/12), low air permeability (0.2 µm/Pa·s), and stronger mechanical properties (4.21 kN/m).	[175]
Taro peel cellulose nanofibers/taro starch	Physical blending: taro starch films with and without cellulose nanofibers were produced by the solvent-casting process.	The addition of cellulose nanofibers caused notable changes in several properties, such as film morphology, thickness, opacity, UV-light barrier capacity, water solubility, and swelling behavior. Films with cellulose nanofiber showed enhanced mechanical features, exhibiting higher Young’s modulus and tensile strength.	[176]
Cellulose nanofiber/starch	Hemiacetal cross-linking: the TCNF suspension (~1% *w*/*v*) and starch pastes (5% *w*/*v*) were blended and stirred until homogeneous dispersions were obtained. The TCNF/starch dispersions were cast in polypropylene Petri dishes and were kept in an oven at 45 °C for 6 h.	Short-term storage of starch pastes was found to improve the mechanical and water resistance properties of TCNF/starch films, owing to the synergistic effect of starch paste crystallinity and hemiacetal cross-linking.	[153]
Pea protein isolate/dialdehyde carboxylated cellulose nanofibers/bilberry extract	Covalently reacting: 10 mL of DCCNFs dispersion was added to the PPI solution (90 mL), and stirring continued for another 30 min. Subsequently, the mixed solution was cooled to 35 °C and then added with BE (1.5 g). The mixture was stirred for another 30 min to obtain a film-forming solution.	DCCNFs and BE were incorporated into the pea protein isolate (PPI)-based films and enhanced the performance of PPI films through the Schiff base reaction. DCCNFs and BE improved the physicochemical properties of the PPI films. The visual color change of the smart film can be used to monitor pork freshness.	[164]
TEMPO-oxidized cellulose nanofiber/starch	Hemiacetal cross-linking: the prepared TCNF dispersion (1.0%, *w*/*v*) and starch solution (3%, *w*/*v*) were blended at a weight ratio of 1.5:1.	Modified starch blending with TCNF enhanced the marine-microbial degradability of TCNF/modified starch film. Higher chemical modification of starch reduced microbial degradability of the film.	[177]

**Table 2:** Selected recent studies on CNF-based composite films with functional materials.

Materials	Preparation	Key Finding	References
CNFs/starch/citric acid	CNFs were dispersed in distilled water using a stirrer at 1000 rpm for 30 min. Starch (3% [*w*/*v*]), glycerol (40% [*v*/*w*]), and CA (0.5% [*w*/*v*]) were added and stirred at 1000 rpm for 5 min for complete dispersion.	Films containing high concentrations of CNF exhibited superior mechanical, thermal, and barrier properties compared to those with low concentrations of CNF and the controls. The coated tomato exhibited a significant reduction in weight loss while retaining the firmness and color of the fruit.	[178]
CNFs/chitosan/zein	For the first layer, chitosan solution was coated on the paper surface. After drying for 12 h at room temperature, MCNF suspension was coated on the surface of single-coated paper as the second coating layer. Zein solution was coated on the surface of dual-layer paper as the third coating layer.	The obtained triple-layer paper had a high kit number of 12 and a low Cobb 60 value of 2.6 g/m^2^ and exhibited heat-sealing ability without any adhesive agent, which has a peel strength of 372.6 N/m. The triple layers also effectively improved the mechanical properties and decreased the moisture and oxygen permeability of the paper substrate. Moreover, the triple-layer paper possesses good antibacterial activity and cytocompatibility.	[179]
Regenerated kenaf CNF/Cur-Zn (II)	To prepare CNF/Cur-metal complex composite films, 5 g of CNF was added to 50 mL of DMAc/LiCl solution. The mixture was stirred until the cellulose solution became clear. Afterward, 5 mg of Cur-Zn (II) complex was added to the CNF solution and continuously stirred.	CNFs derived from kenaf plant were combined with curcumin (Cur)–metal complexes to produce regenerated composite films. Results showed that the Cur-Zn (II) complex-loaded CNF composite films exhibit higher antioxidant activity than other films, whereas Cur-Cu (II) complex-loaded CNF composite films showed prominent antibacterial activity against foodborne pathogenic bacteria such as *Listera monocytogenes* and *Escherichia coli*.	[180]
Soy protein isolate/kappa-carrageenan/CNFs/zenian essential oil	SPI/K-car/BCN (SKB) films were synthesized using the solvent-casting method.	In comparison to the pure SPI film, the film with a high BCN concentration demonstrated a significant decrease in WS (22.98 ± 0.78%), MC (21.72 ± 0.68%), WVP (1.22 ± 0.14 g mm^−1^ S^−1^ Pa^−1^ 10^−10^), and EAB (57.77 ± 5.25%) properties. Zenian-loaded metal-organic frameworks (ZM) substantially enhanced the thermal stability of this film. Furthermore, the ZM films inhibited the growth of pathogenic bacteria and increased the DPPH antioxidant activity.	[181]
chitosan/starch/CNFs/cinnamon essential oil	Nanocomposite films were prepared using the solvent-casting method.	The addition of CEO and cellulose nanofibers was found to enhance the antimicrobial and material properties of the film. The film has also been shown to have antibacterial activity against *Staphylococcus aureus* and *Escherichia coli*.	[182]
Konjac glucomannan/thyme essential oil/bacterial cellulose nanofibers/Ag nanoparticles	KGM powder (0.8%, *w*/*v*) and glycerol 30% (*w*/*w*, based on the weight of KGM) were dissolved in distilled water at 95 °C and stirred for 30 min. BCNs/Ag nanoparticles, TEO (1%, *v*/*v*), and TEO-loaded Pickering emulsions with an oil phase of 10% stabilized by 40 mg BCNs/Ag nanoparticles were added into the basic KGM film-forming solutions (90 mL).	TEO-loaded Pickering emulsions with an oil phase of 10% stabilized by BCNs/Ag nanoparticles films showed the highest contact angle value (86.28°), the best thermal stability and mechanical properties, as well as the best sustained-release property.	[183]
Apple polyphenols/pea starch/pulp cellulose nanofiber	The prepared starch solution (10%, *w*/*v*) was combined with CNF-P aqueous dispersion (0.2%, *w*/*v*) in equal proportions (total volume of 100 mL) and stirred continuously at 86 °C until well mixed. After that, different masses (0.000, 0.025, 0.075, 0.125, 0.175, and 0.225 g) of AP were introduced and blended using magnetic agitation at a temperature of 86 °C.	Apple polyphenols could be uniformly distributed, and form hydrogen bonds with the matrix, and the increase in crystallinity improved the thermal stability of the films (the final residue of the films increased from 22.66% to 31.82%). The TS and EAB of the films reached their maximum values of 11.14 ± 1.73 MPa and 71.55 ± 8.8%, respectively, at an AP content of 1.5%.	[184]
Curcumin/bacterial cellulose nanofiber	The pieces of dried BC nanofiber sheets were soaked in 100 mL of curcumin solution (0.05% (*w*/*v*)) in an acetone-water mixture (ratio of 3:1) for 90 min at 25 °C under gentle stirring (50 rpm).	The curcumin-anthocyanin-loaded nanofiber indicated a distinct color change after spoilage by its exposure to fish meat in a transparent plastic package.	[185]

## 6. Application in Food Preservation

As consumer demand for food freshness and environmental sustainability continues to increase, the functional properties of packaging materials are becoming increasingly important in food preservation applications. CNFs can be effectively used in food packaging applications by blending them with various biopolymers, such as chitosan, starch, or proteins, and incorporating nanomaterials to increase their mechanical strength while reducing permeability to gases and moisture [186]. Sustainably produced CNF films, characterized by excellent mechanical and barrier properties, as well as integrated antimicrobial agents, sensors, and other smart features, are positioned as promising environmentally friendly alternatives to traditional plastics in food packaging solutions.

### 6.1. Application in Fresh Food Preservation

**Figure 4:**
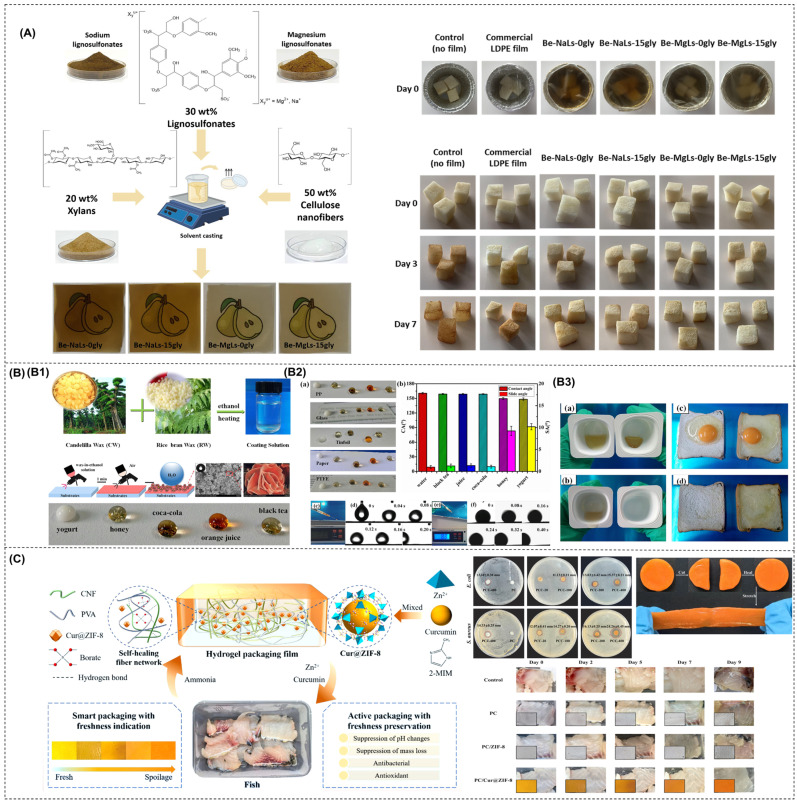
(**A**) Wood-inspired biobased CNF films composed of xylans and lignosulfonates for freshly cut pear preservation [Reproduced from Ref. [200]], (**B**) fabrication of superhydrophobic coatings with CNFs for super-repelling non-Newtonian liquid foods [Reproduced from Ref. [196]], (**C**) CNF-based self-healing hydrogel smart packaging for fish preservation and freshness indication [Reproduced from Ref. [201]].

Ideal food packaging films must achieve a balance between moisture exchange and environmental conditions while effectively protecting food from microorganisms, preventing spoilage, and ensuring safety, thereby playing a crucial role in extending the shelf life. Edible films have been successfully applied to various fresh food categories, including meat [187], seafood [188], fruits [189], and vegetables [190] (Figure 4). For perishable items like fruits and vegetables, maintaining moisture and oxygen balance during storage is vital because their longevity is heavily influenced by moisture loss due to respiration and susceptibility to microbial contamination. Research indicates that regulating the porosity of CNF films can modulate gas exchange, thereby controlling respiration rates and delaying spoilage [191,192]. In the case of meat, fish, and dairy products, films are essential for preventing microbial contamination and maintaining freshness. Fresh meat, a significant source of protein and micronutrients, is particularly vulnerable to microbial contamination and lipid oxidation, which can lead to a decline in nutritional value. Consequently, effective meat packaging must prioritize barrier properties and antimicrobial activity. Xia et al. demonstrated the enhancement of coated paper properties through the incorporation of urethane starch (Sc), calcium lignosulfonate (CL), and CNFs, thereby optimizing electrostatic and hydrogen-bonding interactions. This resulted in an excellent film with hydrophobic, mechanical, gas-barrier, and ultraviolet-blocking properties. Notably, the addition of 0.10% silver nanoparticles (AgNPs) conferred significant antimicrobial activity, extending the shelf life of cherry tomatoes by up to seven days [193]. Zhu et al. used ultrathin cobalt–manganese nanosheets (CoMn-NS) with high specific surface areas grown on pineapple skin CNFs to create a composite (CNF@CoMn-NS) with significant oxidase-like activity. This composite effectively generates reactive oxygen species (ROS), including singlet oxygen (^1^O_2_) and superoxide anions (-O_2_-), and it can eliminate foodborne pathogens such as *Staphylococcus aureus* and *Escherichia coli* without inducing cytotoxicity. The self-assembled CNF@CoMn-NS paper not only exhibits flexibility and stability but also possesses antimicrobial properties that provide significant protection against the wounds of yellow-crowned pears [194]. This strategy effectively prevents decay caused by microbial invasion, thereby improving the preservation of various fruits and vegetables, including pears, dragon fruit, strawberries, and spinach, and significantly extending their shelf life. In recent years, there has been a growing interest in flexible electronics, which requires the integration of electronic components on flexible plastic substrates. CNFs and their derivatives are being explored as potential alternatives to traditional plastics in this area, which could significantly improve food safety in the packaging industry. For example, manufacturers can combat counterfeiting by developing smart packaging solutions that incorporate freshness, temperature, humidity sensors, anti-counterfeiting measures, and interactive features to monitor traceability and supply chain activities [195,196,197]. Zhao et al. developed a ratiometric fluorescent bionanocomposite film containing lignocellulosic cellulose nanofibers (LCNFs) that enhances food freshness detection with high sensitivity (limit of detection (LOD) of 1.83 ppm) and ultra-highly resolvable fluorescence color difference (ΔE = 113.11) for biogenic amines (BA). The performance of the film is attributed to fluorescence resonance energy transfer (FRET), π-π and cation-π interactions between LCNF and fluorescein isothiocyanate (FITC), and a lignin-induced increase in hydrophobicity that enhances amine–FITC interactions. The film exhibited color stability for up to 28 days and a mechanical strength of 49.4 MPa. Freshness monitoring of chicken, shrimp, and balsa fish in conjunction with food freshness indicators yielded consistent and reliable results, making it suitable as an indicator label for commercial plastic packaging. In addition, the researchers developed a smart detection platform using a smartphone for food safety monitoring, enabling the collection of consumption data based on established levels. This innovation represents a rapid and accurate noncontact testing method for food safety monitoring and highlights the commercial potential of CNF-based ratiometric fluorescent biocomposite films [198]. Xu et al. prepared LCNFs with optimized surface properties from bamboo shoot husks. By retaining lignin and using in situ esterification, they achieved ideal wettability, resulting in an LCNF/curcumin Pickering emulsion (CPE) for incorporation into polyvinyl alcohol (PVA) fresh films. The resulting composite film exhibited UV screening, mechanical strength, an oxygen barrier, and antioxidant properties and served as a real-time indicator for monitoring shrimp freshness and maintaining spoilage susceptibility even after six months of storage [199].

### 6.2. Application in Processed Food Preservation

**Figure 5:**
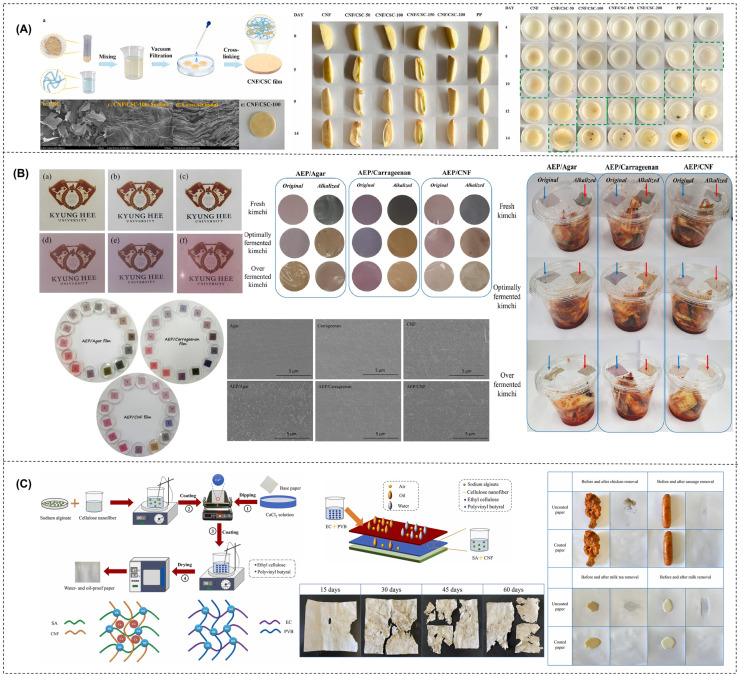
(**A**) Corn straw core/CNF composite for food packaging [Reproduced from Ref. [202]], (**B**) development of smart colorimetric indicators for tracking kimchi freshness by loading aronia extract in agar, κ-carrageenan, and CNF films [Reproduced from Ref. [206]], (**C**) CNF-based self-healing hydrogel smart packaging for fish preservation and freshness indication [Reproduced from Ref. [175]].

Foods, including fruits, vegetables, meats, dairy products, fats, oils, and flours, must be processed and preserved before they can be marketed as ready-to-eat products. The organoleptic properties of foods are critical to consumer satisfaction. Therefore, minimizing changes in nutritional and sensory qualities during processing and preservation is paramount. Effective food preservation is achieved by controlling or inhibiting both external contaminants and internal biological reactions that could affect quality. In food processing, two main strategies are used for preservation: (a) reducing the water activity of the food matrix to inhibit biological reactions and (b) applying heat treatments. CNF films contribute to food protection in two main ways: (a) protecting processed foods from contamination and oxidation and (b) preventing the ingress of water and oxygen, thus maintaining the shape and texture of the products (Figure 5). The inherent heterogeneity of food products requires unique packaging solutions to ensure optimal protection, containment, efficiency, safety, and quality. Perishable liquid foods such as beverages and milk are particularly sensitive to light, oxygen, and microbial contamination. As a result, packaging materials must accommodate liquid processing techniques such as hot filling while ensuring stable product quality. Effective packaging for liquid foods must provide robust gas and moisture barriers and strong mechanical properties, including resistance to breakage and folding. Li et al. developed novel CNF/chitosan (CSC) nanocomposites and compared them with polypropylene (PP), polybutylene adipate copolymer (PBAT), and poly (succinate butylene terephthalate) (PBSeT). The CNF/CSC films exhibited tensile strengths that were 1.25, 4.71, and 8.04 times greater than those of PP, PBAT, and PBSeT, respectively. In addition, their barrier properties were improved by 57.69% and 46.59% compared to CNFs and PBAT, respectively, effectively extending the shelf life of milk by up to 12 days in practical packaging tests [202]. Corn alcohol-soluble proteins covalently linked to CNF films via peptide bonds were used to preserve canola oil. The composite films exhibited significantly lower levels of thiobarbituric acid reactive substances (TBARS) after 14 days of storage at 50 °C compared to control film-preserved canola oil [203]. In the packaging of food products such as bread, biscuits, tea, and coffee, the primary role of CNF films is to provide barrier properties that help maintain the shape and taste of these products. Shih and Zhao developed and characterized starch–CNF biocomposite films that exhibited sufficient water resistance and thermal stability for use in muffin packaging during baking. The incorporation of CNF improved the mechanical properties of cassava and potato films at high temperatures. Specifically, biocomposite films composed of 2% potato starch and 5% CNF showed optimal performance as muffin liners, effectively retaining wet batter and withstanding baking at 177 °C for 20 min, comparable to commercial paper-based muffin liners [204]. The appearance and color attributes of the muffin crust and crumb are illustrated after being packaged by the composite film. Overall, the appearance and color of all muffins were similar based on visual observation. However, the appearance of the liner was significantly (*p* < 0.05) different between paper-based and starch–CNF ones. All the starch–CNF liners were transparent with a slightly white color, while the paper liner was not transparent with a white color. Food films were prepared from guar gum and bagasse-derived CNFs, which showed significant improvements in mechanical and barrier properties. With the incorporation of 0.01 g of CNFs, the films exhibited a significant increase in tensile strength (23.04 MPa), elongation at break (39.19%), tensile modulus (134.94 MPa), and a reduction in water vapor permeability (1.76 × 10^−10^ g m m^−2^ Pa^−1^ s^−1^). In addition, the films exhibited superior resistance to oil permeation, suggesting their potential suitability for packaging oil-based food products [205]. Oun et al. developed color-indicating films containing chokeberry extract powder (AEP) and biopolymers such as agar, carrageenan, and CNFs to monitor the freshness of kimchi. The alkalized AEP/agar films showed pronounced color variations, ranging from greenish-gray (indicative of fresh kimchi, pH 5.5, 0.48% acidity) to light brown (optimal fermentation, pH 4.6, 0.70% acidity) and light purplish-brown (over-fermented, pH 3.80, 1.35% acidity). These alkalized AEP indicator films represent a promising method for real-time detection of freshness in packaged fermented foods such as kimchi [206]. In addition, the color change of the indicator film serves as a non-invasive marker for tracking pH changes in both fresh and aged kimchi without altering the visual appearance of the product. This enables direct, real-time monitoring of product quality throughout storage and transport. Such a system enhances the appeal of packaged goods to consumers by providing them with a visual cue to assess food quality, thus improving transparency and consumer confidence in the freshness and safety of the product.

## 7. Conclusions and Future Perspectives

This paper provides a comprehensive review of the methods used to source, characterize, and functionalize CNFs, with particular emphasis on their applications in active food packaging systems. CNF-based packaging represents a promising basis for the development of green and sustainable food packaging solutions. CNFs significantly improve the mechanical properties of food packaging films through modifications in extraction processes, composite design, and surface treatments. Improved mechanical properties are critical for the large-scale commercial adoption of CNF films. Functionalization—such as surface modification or composite design—significantly improves the barrier properties of CNFs, particularly against water vapor, oxygen, and UV radiation. These advances are critical for extending the shelf life of food products, particularly fresh and perishable items such as fruit, vegetables, and meat, where robust barrier properties can effectively prevent oxidation, spoilage, and moisture loss. Beyond their basic mechanical and barrier properties, significant advances have been made in the functional design of CNF packaging. By incorporating features such as antimicrobial and indicator functions, CNF packaging can not only protect food, but also actively monitor its quality status. This functionalization enhances the competitiveness of CNF packaging in the marketplace and responds to consumer demand for high-performance, intelligent food packaging solutions. In addition, CNFs exhibit excellent biodegradability, making them a more environmentally friendly alternative to conventional plastics, contributing to sustainable packaging initiatives. With the growing emphasis on sustainable development, CNFs offer distinct advantages as packaging materials. As a result, CNFs are increasingly seen as a viable alternative to traditional plastics in the food packaging industry. Despite their wide range of applications and superior performance, several challenges have hindered the transition of CNF films from laboratory research to commercial implementation. These challenges include the following: (a) high production costs, as cellulose’s dense crystalline structure and strong intramolecular and intermolecular hydrogen bonding complicate the manufacturing process, which, despite using agricultural waste, may prevent widespread adoption; (b) unstable yields, due to the variable structure and properties of the CNFs produced, require ongoing research to achieve efficient and reproducible extraction methods; and (c) the processing properties of CNFs films require further improvement, particularly for heat-sealed packaging applications. Addressing these issues is critical to the commercialization of CNF-based packaging. The development of cost-effective and efficient CNF film conversion technologies is key to overcoming these barriers. In addition, the integration of smart packaging, moisture barrier solutions, and 3D printing technologies into food packaging offers significant opportunities to meet evolving market demands. This review provides researchers with an in-depth understanding of the sources, properties, functionalization, and potential applications of CNFs in sustainable food packaging. By optimizing extraction processes, refining functionalization strategies, and improving processing capabilities, CNFs are poised to become a leading material to replace traditional plastics in the food packaging industry, driving the development of more environmentally sustainable packaging solutions.

## Data Availability

No new data were created or analyzed in this study. Data sharing is not applicable to this article.
